# Current use of drains and management of seroma following mastectomy and axillary surgery: results of a United Kingdom national practice survey

**DOI:** 10.1007/s10549-023-07042-7

**Published:** 2023-10-25

**Authors:** K. Fairhurst, K. Roberts, P. Fairbrother, S. Potter, on behalf of the Diamond Study Collaborative, Nick Abbott, Raj Achuthan, Goran Ahmed, Rachel Ainsworth, Laura Arthur, Salena Bains, Zoe Barber, Jeremy Batt, Ashleigh Bell, Jane Carter, Alice Chambers, Anna Conway, Carol-Ann Courtney, Ian Daltrey, Raouf Daoud, Isabella Dash, Rajiv Dave, Julia Dicks, Urszula Donigiewicz, Hiba Fatayer, Daniel Glassman, Nikki Green, Eleanor Gutteridge, Ahmed Hamad, Anita Hargreaves, James Harvey, Shaziya Hassan Ali, Sophie Helme, Julia Henderson, Susan Hignett, Fiona Hoar, Jonathan Horsnell, Thomas Hubbard, Alex Humphreys, Javeria Iqbal, Omotayo Johnson, Meera Joshi, Charlotte Kallaway, Isabella Karat, Baek Kim, Eleftheria Kleidi, Manish Kothari, Chrissie Laban, Kelly Lambert, Siobhan Laws, Alexander Leeper, Serena Ledwidge, Valentina Lefemine, Jonathan Lund, E Jane Macaskill, Mariam Malik, James Mansell, Loaie Maraqa, Yazan Masannat, Julia Massey, Ross McLean, Jennifer McIlhenny, Colin Mcllmunn, Louise Merker, Geraldine Mitchell, Jo Mondani, Elizabeth Morrow, Nabila Nasir, Olubunmi Odofin, Caroline Osborne, Polly Partlett, Anna Powell-Chandler, Sreekumar Sundara Rajan, Clare Rogers, Chandeena Roshanlall, Matthew Philip Rowland, Walid Abou Samra, Lucy Satherley, Brendan Skelly, Richard Sutton, Anne Tansley, Marios Konstantinos Tasoulis, Simon Timbrel, Nader Touqan, Alison Waterworth, Lisa Whisker, Kate Williams, Nihal Gonen Yildirim, Charles Zammit

**Affiliations:** 1 Centre for Surgical Research, Department of Population Health Sciences, Bristol Medical School, University of Bristol, Bristol, England; 2 Bristol Trials Centre, Population Health Sciences, Bristol Medical School, University of Bristol, Bristol, England; 3 Independent Cancer Patient Voice (ICPV), London, England; 4 Centre for Surgical Research, Department of Translational Health Sciences, Bristol Medical School, University of Bristol, Bristol, England

**Keywords:** Mastectomy, Axillary surgery, Drain, Seroma, Trial feasibility

## Abstract

**Purpose:**

Up to 40% of the 56,000 women diagnosed with breast cancer each year in the UK undergo mastectomy. Seroma formation following surgery is common, may delay wound healing, and be uncomfortable or delay the start of adjuvant treatment. Multiple strategies to reduce seroma formation include surgical drains, flap fixation and external compression exist but evidence to support best practice is lacking. We aimed to survey UK breast surgeons to determine current practice to inform the feasibility of undertaking a future trial.

**Methods:**

An online survey was developed and circulated to UK breast surgeons via professional and trainee associations and social media to explore current attitudes to drain use and management of post-operative seroma. Simple descriptive statistics were used to summarise the results.

**Results:**

The majority of surgeons (82/97, 85%) reported using drains either routinely (38, 39%) or in certain circumstances (44, 45%). Other methods for reducing seroma such as flap fixation were less commonly used. Wide variation was reported in the assessment and management of post-operative seromas. Over half (47/91, 52%) of respondents felt there was some uncertainty about drain use after mastectomy and axillary surgery and two-thirds (59/91, 65%) felt that a trial evaluating the use of drains vs no drains after simple breast cancer surgery was needed.

**Conclusions:**

There is a need for a large-scale UK-based RCT to determine if, when and in whom drains are necessary following mastectomy and axillary surgery. This work will inform the design and conduct of a future trial.

**Supplementary Information:**

The online version contains supplementary material available at 10.1007/s10549-023-07042-7.

## Background

Breast cancer affects almost 56,000 women every year in the United Kingdom (UK) [1] and despite improvements in treatment, approximately 40% of these will require mastectomy [2]. Seroma formation following mastectomy and/or axillary clearance is common, with reported incidence in the literature varying between 10 and 85% [3]. Although rarely a serious complication of breast surgery, seroma can cause delayed wound healing, require repeated aspiration with the risk of infection, and may delay the start of adjuvant treatments [4, 5].

Strategies to reduce the formation of seroma include the use of surgical drains and flap fixation methods such as quilting or tissue glue and external compression which all act by minimising the surgical dead-space and evidence to support the effectiveness of different approaches has been summarised in several systematic reviews [6–10]. Many of these reviews, however, have highlighted the lack of high-quality research to support practice and the need for future well-designed studies in this area.

For future research to be meaningful, it is vital that the study design should reflect current practice and address key uncertainties that are important to patients and the clinical community. We aimed to survey breast surgeons to determine current approaches to the management of seroma in the UK; particularly the use of drains after simple breast cancer surgery to inform the feasibility and design of a future randomised controlled trial (RCT).

## Methods

An online national practice survey was developed in REDCap^®^ to capture current UK practice regarding the use of strategies to reduce seroma formation following mastectomy and axillary surgery including the use of drains and flap fixation techniques, and details of the patient pathway for the management of seroma post-operatively. Preliminary work suggested that drains were the most commonly used method of reducing seroma in the UK, so questions focussed on evaluating the feasibility of a future trial comparing the use of drains versus no drains and key elements of trial design including inclusion/exclusion criteria and selection of the primary outcome (Appendix 1).

All consultant breast surgeons and senior breast surgery trainees/fellows, defined as being within their final two years of training, were invited to complete the survey through the professional associations (Association of Breast Surgery (ABS) and the Mammary Fold (UK trainee breast surgery group) and via social media networks. The survey was open for a 4-month period (December 2021–March 2022) and regular reminders were sent to optimise participation. Simple descriptive summary statistics were calculated for each survey item and free text responses were analysed using content analysis [11].

## Results

### Respondent demographics

A total of 147 responses were received of which 97 were completed with data that could be included in the analysis. The partially complete responses were excluded from further analysis where they had very limited, or no data entered. Respondent demographics are summarised in Table 1. Most respondents were consultant breast surgeons (*n* = 75, 77%) with responses received from surgeons practicing across the UK (Table 1).

**Table 1:**
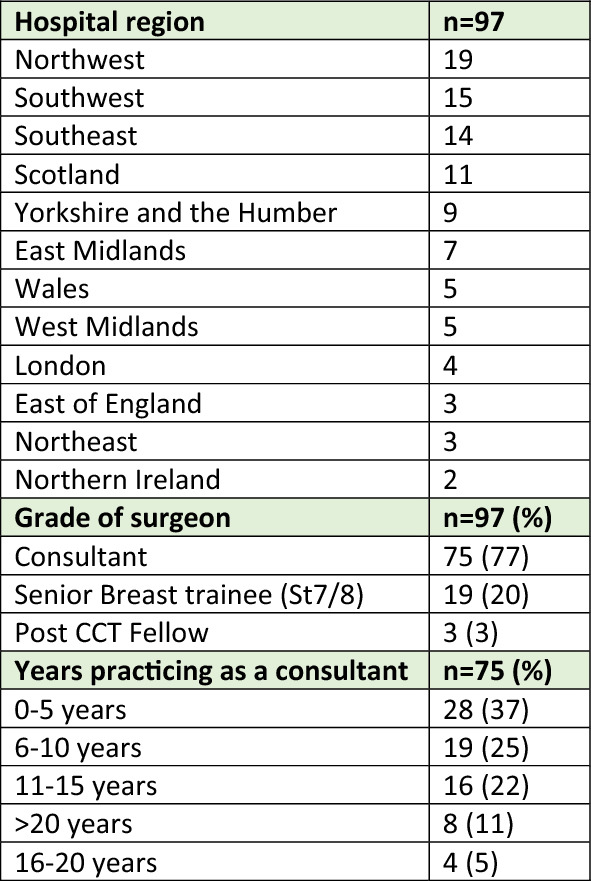
Respondent demographics

### Use of peri-operative interventions to reduce seroma formation

Of the 97 surgeons who completed in the survey, the majority (*n* = 82, 85%) used drains either routinely (*n* = 38, 39%) or in certain circumstances (*n* = 44, 45%). This was most frequently a single drain, although when mastectomy was combined with an axillary node clearance (ANC), two drains were used by a proportion of surgeons (Table 2).

**Table 2:**
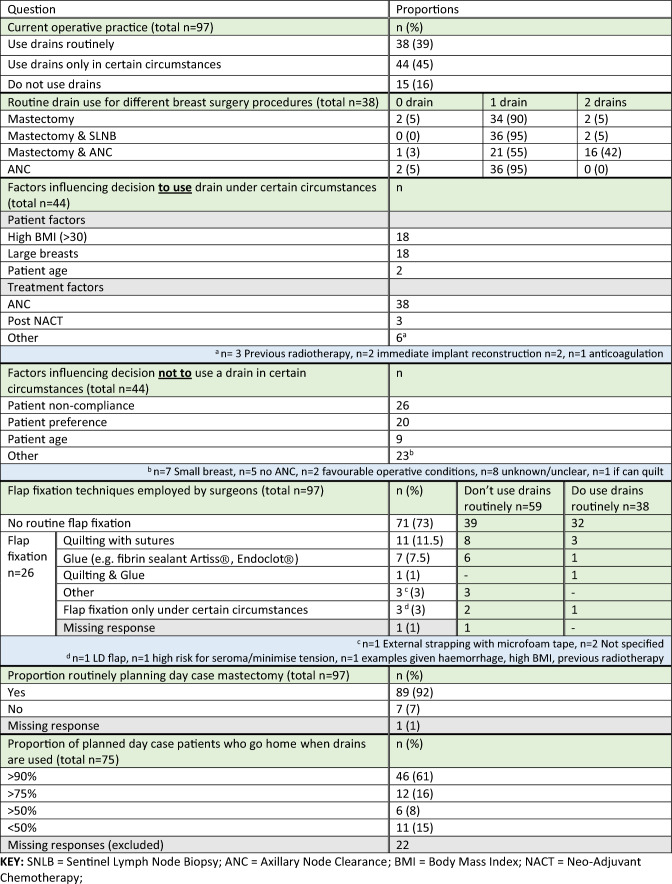
Operative practice regarding drain use following mastectomy and axillary surgery

The indications for selective drain use included patient (e.g. age, high body mass index or large breasts) and treatment factors (e.g. extent of surgery or after neo-adjuvant chemotherapy). The most common reasons for not using a drain included non-compliance and patient preference (Table 2). Whilst further details on how patient preference influences drain use were not collected by this survey, drain placement is part of the informed consent process and some patients may therefore decline drain insertion despite the reasons for use being explained.

Few surgeons (*n* = 26/97, 27%) reported routinely using flap fixation methods to reduce seroma. The most commonly used methods were quilting (*n* = 11, 11.5%) and glue sealants (*n* = 8, 8.5%). These methods were more frequently used by surgeons who did not routinely use drains (*n* = 20, 34% vs *n* = 6, 16%) (Table 2).

### Post-operative patient pathway

Significant variability in the post-operative management of drains and seromas was reported. Of the 79 respondents using drains, the majority (*n* = 59, 62%) removed them based on the volume of seroma drained per day, most commonly < 50 mls/24 h (Table 3). Drains were most frequently removed by nursing staff (*n* = 63/106, 60%) in breast clinic (*n* = 44, 56%).

**Table 3:**
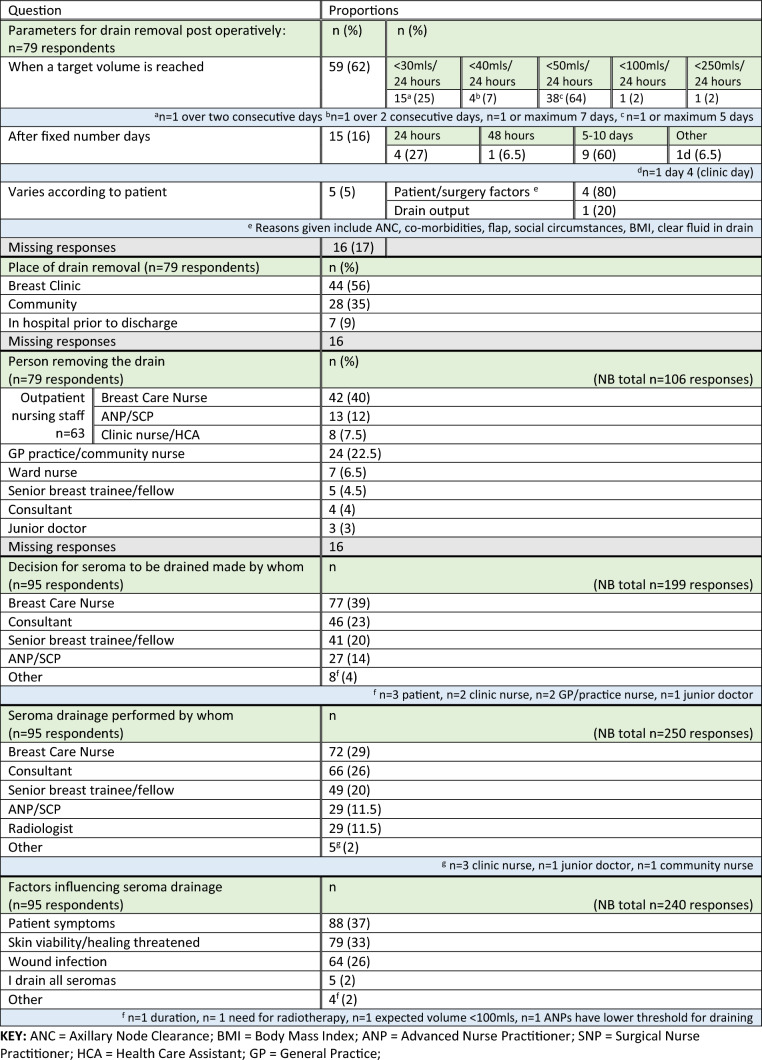
Post-operative patient pathway of care regarding drain removal following mastectomy and/or axillary surgery (*n* = 95 survey participant responses)

Breast care nurses were most frequently responsible for assessing patients; deciding when seromas should be drained (77/199, 39%) and performing the procedure (72/250, 29%). Factors influencing decision-making regarding seroma drainage included patient symptoms (88/240, 37%) and assessment of actual (e.g. infection, 64/250, 26%) or impending (e.g. concerns re skin viability, 79/250, 33%) complications. Very few respondents reported draining all seromas (Table 3).

### Feasibility and design of a future RCT

Of the 91 surgeons completing this section of the survey, just under half (*n* = 37, 41%) expressed uncertainty regarding the use of drains after routine breast cancer surgery. Of those indicating some uncertainty, this was mostly regarding the use of a drain following mastectomy and sentinel lymph node biopsy (SLNB) (*n* = 33, 70%) and isolated ANC (*n* = 29, 62%). Two-thirds of surgeons felt a trial comparing the use of drains vs no drains after simple breast cancer surgery was needed (n = 59, 65%).

Almost half of the surveyed surgeons (*n* = 45, 49%) indicated they would be willing to randomise all patients undergoing mastectomy ± axillary surgery in an RCT comparing the use of drains versus no drains. The remaining half expressed reluctance randomising specific groups of patients. These groups primarily included patients in whom the surgical dead-space was anticipated to be large, for example following mastectomy and ANC (29/83, 35%); in women with Body Mass Index (BMI) > 30 (16/83, 19%); in those with large breasts (12/83, 15%) or; in those perceived to be at higher risk of post-operative complications (13/83, 16% e.g. those with high risk of bleeding; post neo-adjuvant chemotherapy etc.) (Table 4).

**Table 4:**
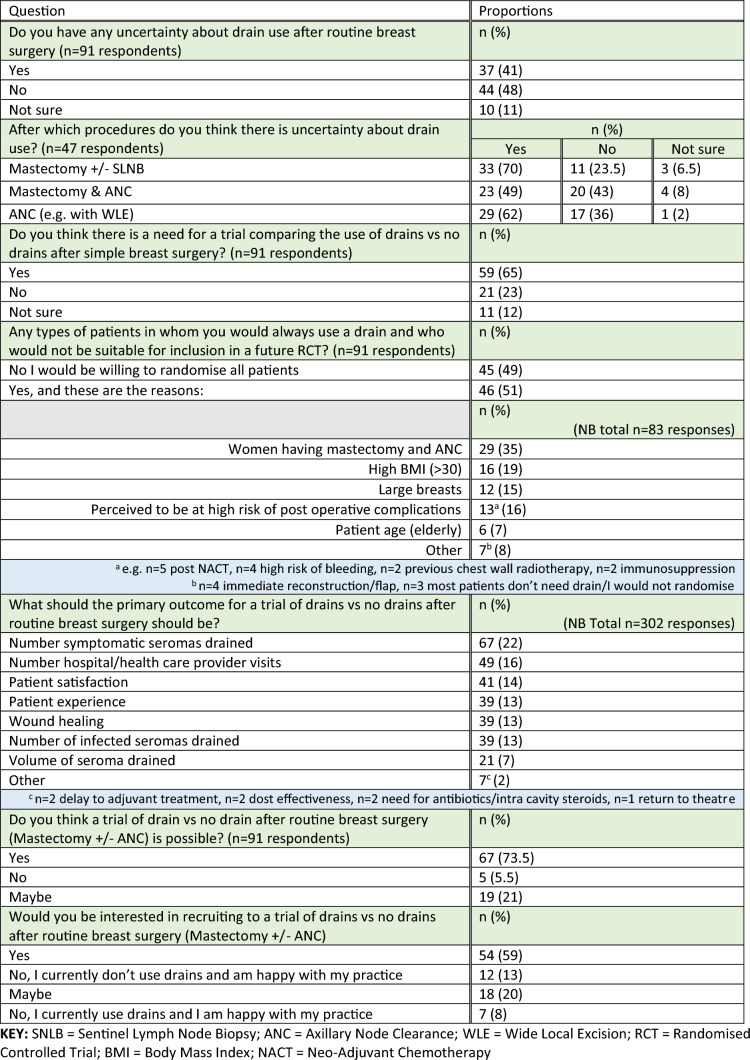
Feasibility of a trial examining comparing drains vs no drains following mastectomy and/or axillary surgery (*n* = 91 responses)

There was a lack of consensus regarding the most suitable primary outcome for a future trial. Respondents were asked to select all outcomes of importance and/or interest in order to try and elucidate whether a single or co-primary endpoint might be necessary for a future trial. The most commonly selected outcome was the number of symptomatic seromas drained (67/302, 22%), followed by the number of hospital/healthcare provider visits (49/302, 16%) and patient reported outcomes such as patient satisfaction (*n* = 41/302, 14%) (Table 4). Almost three-quarters (*n* = 67, 73.5%) of respondents felt that a future trial was feasible and almost 80% would be definitely (*n* = 54) or possibly (*n* = 18) interested in recruiting patients to a future RCT.

Of 91 respondents, 52 (57%) provided free text comments relating to the feasibility and design of a future trial. Three key themes emerged (Table 5) from the content analysis. These were: 1. The need for high-quality evidence to inform, change and address variation in practice (specifically no longer using drains); 2. Clinical (personal and community) equipoise and; 3. Trial design, outcome selection and feasibility. Overall, respondents felt that as there was significant variability in UK practice and no clear evidence to support the use of drains, a trial was necessary.

**Table 5:** Key themes emerging from content analysis of free text survey responses

Theme	Survey respondent quotes	
The need for high-quality evidence to inform best practice	Respondent 71: “To provide evidence regarding the indications and benefits of drain use where it is currently lacking. The avoidance of drains would offer an additional economic benefit.” Respondent 69: “It would be very useful for our hospital bed capacity if we were confident that no drain use was safe and these 1–2 night stay patients could be considered for day case surgery.” Respondent 66: “In my own practice I am satisfied that the seroma and wound complication rate without drains is lower than with drains, and has significantly lower patient discomfort. I am not sure the results of a randomised trial would be applicable to my own technique and would be unlikely to change practice, however a trial may be necessary for drains to be abandoned as a routine.” Respondent 58: “…i think it is much less certain whether drains are required after an axillary node dissection and for my own practice a trial would be helpful to guide clinical practice”	
Equipoise	Personal equipoise	Respondent 19: “I really do not know if drains are beneficial but I am also unsure if not using them is the 'right' thing to do. I would very much support an RCT” Respondent 83: “I would like confirmation of my assumption that drains are not helpful”
	Lack of equipoise	Respondent 34: “Not sure I'm willing to go back to using drains” Respondent 37: “May be difficult to establish equipoise. A place I worked in trialled stopping using drains, but then felt they had more problematic haematomas/seromas so went back to using drains. They are unlikely to want to stop using drains.”
	In equipoise in some settings	Respondent 139: “I am comfortable that in my practice drains are not needed for the vast majority of patients but there may be a subset where their use is indicated such as in obese patients.”
Trial design and feasibility	Respondent 133: “simple intervention, no high-quality evidence either way, high volume procedures” Respondent 35: “Long overdue study. Would be easy to instigate but practice does seem to vary between consultants” Respondent 42: “It's a good question that needs studying and there are enough surgeons wanting to answer the question.” Respondent 103: “There are units who routinely use drains and others who doesn’t. So it should be possible to randomize and accrue sufficient number of people to achieve adequate power to detect a difference.” Respondent 110: “Because there is currently no consensus and patients will generally be guided by surgeons on this so I would expect good compliance and no issues with recruitment numbers” Respondent 83: “There is variation in practice so lack of consensus in surgical community. Patients would benefit and question is clear and so would not be difficult to recruit to” Respondent 82: “Any trial would be valuable but I feel it is important for the data to capture how many times a patient WITHOUT any drain contacts and/or attends the breast clinic for seroma drainage or wound check, and if they perceive a less comfortable QOL existence in the first 1–2 weeks after surgery when seroma volume is maximal.” Respondent 43: “I think no drain policy may increase load on BCN and surgeons for seroma aspiration	

## Discussion

This survey has demonstrated considerable variation in the management of seromas following mastectomy and axillary surgery in the UK and the need for and potential feasibility of a large-scale pragmatic RCT to establish best practice. It is likely that a future trial would compare the use of drains vs no drains as this is currently the main strategy for reducing seroma development in the UK.

Surgeons’ attitudes to a potential trial in this area reflects the lack of high-quality evidence to support the use of drains following breast cancer surgery. A systematic review [6] considered seroma formation in 1347 women following mastectomy (± axillary lymph node clearance) with and without suction drainage. The review included two RCTs [12, 13] and six non-randomised studies [14–19]. The data were found to be at a high risk of bias, heterogeneous with variable use of flap fixation methods and with an inconsistently defined primary outcome of seroma formation. The authors concluded that there was some evidence that drainage following mastectomy and axillary surgery could be safely omitted without increasing seroma formation or complications but highlighted the need for further high-quality research to determine the role of surgical drains following breast cancer surgery including outcomes of importance to patients. These findings were consistent with previous reviews [9, 20, 21] suggesting that drainage does appear to reduce seroma rates but may be associated with longer hospital stays. However, it should be noted that drain use increasingly may not affect hospital stay as significantly as it has done in the past. The 2021 Getting It Right First Time (GIRFT) report (Using Hospital Episode Statistics, HES Data April 2015-March 2018) [22], demonstrated that only 20% of mastectomies without reconstruction, were conducted as a day case and that rates vary widely across trusts from 0% to 78.28%. The report recommended that day case mastectomy rates should be increased to 75%. Increasing day case mastectomy has perhaps recently been driven by the COVID-19 pandemic, and the consequent need to avoid hospital stay and risk of infection. In this survey, 77% (58/75) of respondents reported that patients went home on the day of surgery more than 75% of the time (Table 2).

Several recent or ongoing European RCTs, as well as comprehensive literature reviews [23] have considered techniques to reduce seroma formation and the need for drains after mastectomy. The Dutch SAM trial [24, 25] (NCT03305757), was a multicentre three arm RCT of flap fixation with sutures or tissue glue and conventional closure, with closed suction drains in all arms. This showed a significant reduction in clinically significant seroma in both flap fixation arms with the greatest reduction in the sutured flap fixation group [24]. Ongoing RCTs include the single-centre Dutch SARA [26] (NCT04035590) trial which will compare flap fixation with and without suction drainage; the multicentre Dutch QUILT (NCT05272904—not yet recruiting) trial comparing quilting without a drain and conventional closure; and the multicentre French QUISERMAS trial [27] (NCT02263651) comparing conventional closure with a drain and flap fixation without a drain. This study completed in 2018 but has yet to report. None of these trials, however, reflect current UK practice or include outcomes of importance of patients. Quilting is not standard practice in the UK, perhaps due to the increased costs associated with the time quilting takes and the use of more expensive self-locking sutures to perform the procedure. In addition, there are perceived concerns regarding compromising skin flap viability, particularly where the skin flaps are thin or in those already deemed to be high risk for complications such as smokers.

Whilst the most common outcome for the trial suggested by surgeons in this survey was the number of symptomatic seromas drained (20% of respondents), this was closely followed by the number of hospital/healthcare provider visits (16% of respondents). Work with our patient and public involvement (PPI) group highlighted that hospital visits were perceived as a major burden to patients. This outcome would comprehensively evaluate drain-related issues, symptomatic seromas; wound complications and patient concerns which may require clinical evaluation while being objective and easy to measure. As such, hospital visits would pragmatically be the most appropriate primary outcome for a future trial.

This is a national practice survey with limitations that require consideration. Firstly, it only includes the views of a relatively small group of UK breast surgeons. From the Getting It Right First Time (GIRFT) report in 2021 [22], there were 130 breast surgery units in England, but this number varies year to year depending on service mergers and closures. The surgeons who responded, may be more engaged in research and thus may not be representative of the breast surgical community more broadly. Whilst this is possible, the survey has included surgeons from across the UK, in all major geographical areas, with various degrees of experience. Furthermore, this engaged group of surgeons is likely to include those who will open and recruit to any future study. It could therefore be argued that their views are the most relevant as they will determine whether a future study would be successful. It is, however, possible that willing to participate in a future RCT in principle, does not always translate into actual participation in practice.

Despite limitations, this work demonstrates there is a need for a high-quality RCT to determine if, when and in whom closed suction drains are necessary following mastectomy and axillary surgery in the UK. Perhaps more importantly, this is a question that is also meaningful to patients as in the recent James Lind Priority Setting Partnership (PSP) in breast cancer surgery [28], one in three patient respondents submitted questions related to seroma and the benefits of drains after breast cancer surgery. Overall, this question was ranked as the 11^th^ most important research priority to patients completing the survey and although it narrowly missed being considered for the top 10 research priorities [28], it is clearly an area where more research is needed.

Work to design and gain funding for a future trial is now underway. Given the large volume of procedures performed, it is likely that that such a trial would recruit quickly and easily and utilisation of the breast trainee collaborative research network may represent a cost-effective means of delivering the study in a timely fashion [29–31]. If an RCT proves that drains are unnecessary in all or most patients undergoing mastectomy and/or axillary surgery, it will provide the necessary high-quality evidence to change practice. This will reduce NHS costs and the burden on scarce resources, but more importantly, improve patient experiences of breast cancer treatment.

### Supplementary Information

Below is the link to the electronic supplementary material.

Supplementary file1 (DOCX 215 KB)

## Data Availability

The datasets generated and analysed during this study are stored under the provisions of the National Data Protection Act and the University of Bristol requirements. Data may be made available to bona fida researchers only, on reasonable request to the corresponding author, after their host institution has signed a Data Access Agreement.
