# Supplementary material for: Current use of drains and management of seroma following mastectomy and axillary surgery: results of a United Kingdom national practice survey
Source: Breast Cancer Res Treat. 2023 Oct 25;203(2):187–96. doi: 10.1007/s10549-023-07042-7 (PMC10787912; doi:10.1007/s10549-023-07042-7)

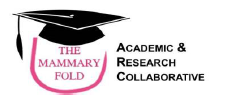

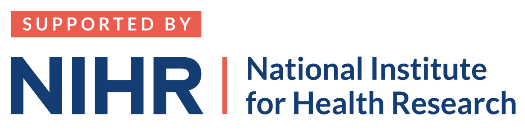

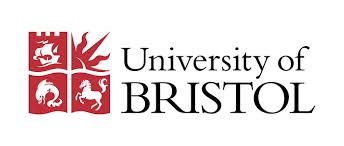


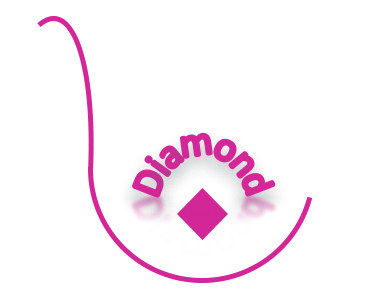


**Drains vs no drains after mastectomy and axillary surgery**

Drains have routinely been used for many years following breast surgery to reduce seroma formation, and to prevent potential wound healing problems that may delay adjuvant treatments. However, drain(s) after breast surgery may cause anxiety, inconvenience and discomfort for many women. The use of drains may also prevent same-day discharge from hospital (daycase surgery) due to a lack of patient confidence in managing the drain(s).

While drain use continues to be routine practice in many units throughout the UK, there is no high-quality evidence to support the true benefits of using drains for reducing seroma and post-operative complications.

The Mammary Fold Academic & Research Collaborative are planning to design and deliver a large-scale pragmatic RCT comparing the use of drains vs no drains in women undergoing mastectomy and/or axillary surgery in the UK, to determine whether drain use should continue. It is important that this study is well-designed to reflect current practice and any uncertainties within the surgical community.

If you are a consultant or senior breast trainee (ST7+) we would therefore be very grateful if you would complete this short 10-minute survey regarding your current practice and attitudes to a future definitive trial. Everyone who completes the survey will be listed as a collaborator on any publications. All responses will be entirely anonymous.

Many thanks in advance for your time and effort in completing this survey.

***Part one: Surgeon demographics***

1. Location
   1. Which **hospital** in the UK do you currently work in?

Free text:

- 1. Which **region** of the UK is this hospital in?
     1. Scotland
     2. Northern Ireland
     3. Wales
     4. North East
     5. North West
     6. Yorkshire and the Humber
     7. East Midlands
     8. West Midlands
     9. South West
     10. South East
     11. London
     12. East of England

1. What **grade** of breast surgeon are you?
   1. Consultant Breast Surgeon
   2. ST7/8 Breast trainee
   3. Post CCT Fellow
2. If you are a consultant, for **how many years** have you been practicing?
   1. Not applicable
   2. 0-5
   3. 5-10
   4. 10-15
   5. 15-20
   6. >20
3. Name (for citation purposes only):

***Part two: Current Operative Practice***

1. Do you **routinely use a drain** following?
   1. Mastectomy only
      1. None
      2. One
      3. Two
   2. Mastectomy and sentinel node biopsy
      1. None
      2. One
      3. Two
   3. Mastectomy and axillary node clearance
      1. None
      2. One
      3. Two
   4. Axillary node clearance
      1. None
      2. One
   5. I only use a drain under certain circumstances (see question 6)
2. If you **only use a drain under certain circumstances**, what factors influence your decision to:
   1. **Use** a drain? (Please check all that apply)
      1. Patient age (Elderly)
      2. High BMI (>30)
      3. Large breasts
      4. Women having mastectomy with axillary node clearance
      5. Post neoadjuvant chemotherapy
      6. Other (Please give details):
   2. **Not use** a drain? (Please check all that apply)
      1. Patient non-compliance (e.g. cognitive impairment or mental health issues)
      2. Patient preference/circumstances (e.g. young children)
      3. Patient age (Young)
      4. Other (Please give details):
3. If you **don’t** use drains routinely after mastectomy, do you use any of the following flap fixation methods? (Please check all that apply)
   1. I don’t routinely perform any flap fixation
   2. Quilting with sutures
   3. Glue
   4. Other (Please give details):
   5. I perform flap fixation only under certain circumstances (Please list):

1. If you **do** use drains routinely after mastectomy, do you use any of the following flap fixation methods **in addition to drains**? (Please check all that apply)
   1. I don’t routinely perform any flap fixation
   2. Quilting with sutures
   3. Glue
   4. Other (Please give details):
   5. I perform flap fixation only under certain circumstances (Please list):
2. Do you routinely plan to perform **day case mastectomy** in suitable patients?
   1. Yes
   2. No
3. If you use a drain(s), approximately **what proportion** of your planned day case mastectomy patients go home on the same day as surgery?
   1. >90%
   2. >75%
   3. >50%
   4. <50%

***Part three: Current Post-Operative Practice & Pathway of Care***

1. When you use a drain, what **parameters** do you use **to guide removal** of the drain(s)?
2. After a fixed number of days
   - 1. 24 hours
     2. 48 hours
     3. 3 days
     4. 5-10 days
     5. Other (Please specify):
     6. Not applicable – I don’t use drains
3. When a target drain volume is achieved
   1. <30mls over 24 hours
   2. <50mls over 24 hours
   3. Other (Please specify):
   4. Not applicable – I don’t use drains
4. Varies according to patient (Please specify):
5. **Where** are the drains usually removed?
   1. In the breast clinic
   2. In the community
   3. Other (Please specify where):
   4. Not applicable – I don’t use drains
6. **Who** normally removes the drains? (Please check all that apply)
   1. Breast care nurse
   2. Advanced Nurse Practioner (ANP) or Surgical Care Practioner (SCP)
   3. Senior breast surgery trainee/fellow
   4. Radiologist
   5. Consultant
   6. Other (Please specify who):
   7. Not applicable – I don’t use drains
7. When a patient reports that they have developed a seroma, **who routinely reviews the patient and decides** whether the seroma should be drained? (Please check all that apply)
   1. Breast care nurse
   2. Advanced Nurse Practioner (ANP) or Surgical Care Practioner (SCP)
   3. Senior breast surgery trainee/fellow
   4. Radiologist
   5. Consultant
   6. Patient
   7. Other (Please specify who):
8. What factors **influence your decision** to drain a seroma? (Please check all that apply):
   1. Evidence of wound infection
   2. Skin viability/wound healing threatened
   3. Patient symptoms (e.g. discomfort/pressure, leaking)
   4. I drain all seromas
   5. Other (Please specify):
9. **Who** performs seroma drainage in your practice? (Please check all that apply)
   1. Breast Care Nurse
   2. Advanced Nurse Practioner (ANP) or Surgical Care Practioner (SCP)
   3. Registrar or Senior breast surgery trainee/fellow
   4. Radiologist
   5. Consultant
   6. Other (Please specify who):

***Part four: A potential randomised trial of drain vs no drain following mastectomy and axillary surgery***

1. Do you have any **uncertainty** about whether drains should be used after routine oncological breast surgery (simple mastectomy and/or axillary clearance)?
2. Yes
3. No
4. Not sure
5. After **what procedures** do you think there is **uncertainty** about whether drains should be used?
   1. Simple mastectomy and/or sentinel lymph node biopsy
      1. Yes
      2. No
      3. Not sure
   2. Mastectomy and axillary clearance
   3. Yes
   4. No
   5. Not sure
   6. Axillary clearance (e.g. with wide local excision)
      1. Yes
      2. No
      3. Not sure
6. Do you think there is **a need for a trial** comparing the use of drains vs no drains after simple breast surgery?
   1. Yes
   2. No
   3. Not sure
   4. Please give details:
7. Do you think a trial of drain vs no drain following mastectomy and/or axillary surgery is **possible**?
   1. Yes
   2. No
   3. Maybe
   4. Why do you think this? Please comment:
8. Would there be any types of patients in whom you would always use a drain and who **wouldn’t be suitable for inclusion** in a future randomised study? (Please check all that apply)
   1. No, I would be willing to randomise all patients
   2. Patient age (Elderly)
   3. High BMI (>30)
   4. Large breasts
   5. Women having mastectomy with axillary node clearance
   6. Post neoadjuvant chemotherapy
   7. Other (please specify):
9. What do you think the **primary outcome** of a future trial comparing drains vs no drains should be?
10. Number of seromas drained
11. Volume of seroma drained
12. Number of hospital visits
13. Wound healing
14. Patient satisfaction
15. Patient experience
16. Other (please specify):
17. Would you be interested in **recruiting to** a trial comparing the use of drains vs no drains following mastectomy and/or axillary surgery?
18. Yes
19. No - I currently don’t use drains and am happy with my practice
20. No - I currently use drains and am happy with my practice
21. Maybe
22. If you are interested in participating in a future trial, **please fill in your contact details here** (note your details will only be used to contact you about the **Diamond** study)
    1. Name
    2. Email Address

**Many thanks for taking part**

***Kit Fairhurst***

***& Shelley Potter***

**on behalf of the Diamond study team**


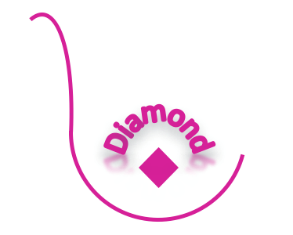

Supplement: Supplementary file 1 — Supplementary file1 (DOCX 215 KB) [file 10549_2023_7042_MOESM1_ESM.docx]
